# A Network of HSPG Core Proteins and HS Modifying Enzymes Regulates Netrin-Dependent Guidance of D-Type Motor Neurons in *Caenorhabditis elegans*

**DOI:** 10.1371/journal.pone.0074908

**Published:** 2013-09-16

**Authors:** Stephan Gysi, Christa Rhiner, Stephane Flibotte, Donald G. Moerman, Michael O. Hengartner

**Affiliations:** 1 Institute of Molecular Life Sciences, University of Zurich, Zurich, Switzerland; 2 Neuroscience Center Zurich, University of Zurich, Zurich, Switzerland; 3 Institute of Cell Biology, University of Bern, Bern, Switzerland; 4 Department of Zoology, University of British Columbia, Vancouver, Canada; Columbia University, United States of America

## Abstract

Heparan sulfate proteoglycans (HSPGs) are proteins with long covalently attached sugar side chains of the heparan sulfate (HS) type. Depending on the cellular context HS chains carry multiple structural modifications such as sulfate residues or epimerized sugars allowing them to bind to a wide range of molecules. HSPGs have been found to play extremely diverse roles in animal development and were shown to interact with certain axon guidance molecules. In this study we describe the role of the *Caenorhabditis elegans* HSPG core proteins Syndecan (SDN-1) and Glypican (LON-2) and the HS modifying enzymes in the dorsal guidance of D-type motor axons, a process controlled mainly by the conserved axon guidance molecule UNC-6/Netrin. Our genetic analysis established the specific HS code relevant for this axon guidance event. Using two sensitized genetic backgrounds, we isolated novel components influencing D-type motor axon guidance with a link to HSPGs, as well as new alleles of several previously characterized axon guidance genes. Interestingly, the dorsal axon guidance defects induced by mutations in *zfp-1* or *lin-35* depended on the transgene *oxIs12* used to visualize the D-type motor neurons. *oxIs12* is a large multi-copy transgene that enlarges the X chromosome by approximately 20%. In a search for genes with a comparable phenotype we found that a mutation in the known dosage compensation gene *dpy-21* showed similar axon guidance defects as *zfp-1* or *lin-35* mutants. Thus, derepression of genes on X, where many genes relevant for HS dependent axon guidance are located, might also influence axon guidance of D-type motor neurons.

## Introduction

Extension and guidance of axons during nervous system development rely on a number of conserved axon guidance cues [1]. In addition, there is increasing evidence that other systems fine-tune the known major axon guidance cue families. Heparan Sulfate Proteoglycans (HSPGs) have been shown to encode specific information for the development of nervous systems through specifically modified covalently attached carbohydrate polymers, the glycosaminoglycan (GAG) heparan sulfate (HS) [2,3,4,5,6]. The core proteins can be grouped into two classes, (i) membrane associated core proteins, such as the transmembrane Syndecans (*sdn-1* in *C. elegans*) and the glycosylphosphoinositol (GPI) anchored Glypicans (*gpn-1, lon-2*) and (ii) core proteins of the extra cellular matrix (ECM), which include Perlecan (*unc-52*), Agrin (*agr-1*) and Collagen XVIII (*cle-1*) (reviewed in 7,8,9). Studies in various organisms including worms, flies and mammals have shown that HSPGs play diverse roles in the nervous system (reviewed in 10). However, studying HSPGs in mammals is challenging because most members are present in multiple copies and are most probably functionally redundant.

While the core proteins for HS chains are responsible for some diversity of HSPGs in different cell types, the largest source of diversity comes from variable modifications of heparan-sulfate disaccharides, including the epimerization of the fifth carbon atom in hexuronic acid and sulfation at several sites. Similar to the core proteins, the genes coding for the enzymes catalyzing the modifications are well conserved in *C. elegans* [11]. Deacetylation is performed by *hst-1*, which is an essential gene in the worm, probably due to the fact that deacetylation precedes and paves the way for all sulfations. HS chains in *hst-1* mutants are most likely entirely unsulfated and therefore lack any specificity. 2-O, 3-O, and 6-O sulfations are catalyzed by *hst-2*, *hst-3.1* and *hst-3.2*, and *hst-6*, respectively. The gene for C5 epimerization is *hse-5*. Finally, the HS chains are about 100 or more sugar units long and have numerous structural heterogeneities offering the possibility to interact with a large number of ligands [12,13,14,15]. The large negative charge of the HS chains is a result of sulfation and is one reason why many molecules bind HS. The modifications are complex and incomplete, giving rise to an enormous number of possible modification patterns [16,17]. A core protein can therefore carry different context dependent modifications, resulting in an HS code that is required on a specific neuron or in its environment to modulate certain axon guidance choices [9,16,18].

Across all species, populations of axons project towards or away from the midline of the nervous system. Members of the highly conserved Netrin family of proteins mainly mediate this attraction and repulsion. The Netrins are secreted guidance molecules with one known member in *C. elegans* (UNC-6 [19]). Also, the receptor mechanisms for Netrin are highly conserved throughout the animal kingdom. Growth cone attraction is triggered by the binding of UNC-6/Netrin to homodimers of UNC-40/deleted in colorectal cancer (DCC) receptor family proteins, while growth cone repulsion is mediated by the UNC-5 family of receptor proteins, in some cases together with UNC-40 [20], (reviewed by [21]). In the worm, UNC-5, UNC-6 and UNC-40 represent the major system for circumferential guidance of migrating cells and axons towards and away from the ventral nerve cord (VNC) [19].

The major classes of motor neurons in *C. elegans* (DA, DB, DD, VD and AS motor neurons) have cell bodies in the VNC and send commissural axons to the dorsal nerve cord (DNC). UNC-6 is expressed ventrally to form a gradient. UNC-5 and UNC-40 are expressed in dorsally migrating axons to mediate a repulsive response to UNC-6, whereas UNC-40 is expressed in ventrally migrating axons to mediate an attractive response to UNC-6 [22,23].

In addition to UNC-6 the dorsally expressed UNC-129/TGF-β family ligand that establishes a gradient opposite to UNC-6 also plays an important role in guidance of dorsally migrating axons [24]. Axons growing out from the ventral side of the worm first use the UNC-5 receptor to respond to high concentrations of UNC-6. As the growth cone moves further away from the ventral side the concentration of UNC-6 decreases while the concentration of UNC-129 increases. UNC-129 binds to UNC-5 and is thought to induce a switch in UNC-6 signaling from the UNC-5 only signaling to UNC-5 + UNC-40 signaling, which is more sensitive to UNC-6 and can therefore maintain the repulsive effect of UNC-6 even in areas where the UNC-6 concentration is very low [24]. In *Drosophila* the establishment of the Dpp gradient, a TGF-β homolog was shown to depend on HSPGs [25]. What role HSPGs play in the integration of the UNC-6 and UNC-129 gradients in *C. elegans* is an open question.

In mice, axons growing towards the floor plate in the spinal chord fail to respond to the attractive Netrin signal secreted by the floor plate if HSPGs lack functional HS side chains [26], suggesting that HSPG function is vital for proper Netrin signaling. We therefore hypothesized that a systematic study of the HSPG network required for ventral to dorsal axon guidance in *C. elegans* would provide the possibility to establish a clearer link between Netrin and HSPGs and to define the HS code relevant for Netrin signaling. In this study we show that the HSPG network guiding D-type motor axons is comprised of two parallel pathways containing either SDN-1 or LON-2 as core protein. In an enhancer screen we find new components influencing D-type motor axon guidance and show that Netrin signaling through the UNC-5 receptor could depend on HSPGs. Furthermore, our data indicate that derepression of genes located on the X chromosome influences axon guidance, most likely because many genes relevant for D-type motor axon guidance are located on X.

## Materials and Methods

### Strains, general handling and procedures

Standard methods were used for maintenance and handling of *C. elegans* as described by 27. Briefly, worms were grown on *E. coli* (strain OP*50*) and the *C. elegans* variety Bristol N2 was used as wild type and kept at 20° if not stated otherwise. The Hawaii variety (CB4856) was used for mapping of mutations.

The following mutations and transgenes were used:

LGI: *lin-35*(*n745*) [28], *oxIs268*[*unc-47::gfp*] [29], *cle-1*(*cg120*) [30], *unc-73*(*op464*) (this study).

LGII: *ptp-3*(*op147*) [31], *ptp-3*(*ok244*)*, unc-52*(*e444*) [32], *unc-53*(*ok2736*)*, unc-53*(*op479*) (this study), *unc-130*(*oy10*) [33], *unc-130*(*op459*) (this study), *juIs76*[*unc-25::gfp; lin-15(+*)] [34], *hst-3.1*(*ok1249*) [35], *hst-3.1*(*tm734*) [35].

LGIII: *hse-5*(*tm472*) [16], *zfp-1*(*ok554*) [36], *zfp-1*(*op481*) (this study).

LGIV: *eri-1*(*mg366*) [37], *unc-129*(*ok1443*)*, unc-5*(*e53*) [27], *unc-5*(*op460*)*, unc-5*(*op468*)*, unc-5*(*op469*)*, unc-5*(*op477*) (all this study), *zag-1*(*op462*) (this study).

LGV: *oyIs14*[*sra-6::gfp; lin-15(+*)] (Piali Sengupta), *max-1*(*op482*) (this study), *dpy-21*(*e428*) [38].

LGX: *lon-2*(*e678*) [27], *hst-6*(*ok273*) [16], *sdn-1*(*zh20*) [5], *hst-2*(*ok595*) [16], *gpn-1*(*tm588*) [39], *oxIs12*[*unc-47::gfp; lin-15(+*)] [40], *hst-3.2*(*tm3208*) [35], *hst-3.2*(*tm3006*) [35], *pkIs296*[*hsp::gsa-1(Q208L*)*; dpy-20(+*)] [41].

### Ethyl Methanesulfonate (EMS) Enhancer screens

For mutagenesis, staged *hse-5*(*tm472*) or *sdn-1*(*zh20*) mutant L4 animals carrying the *oxIs12* transgene were mutagenized with ethyl methanesulfonate (EMS) as described by 27 and distributed onto four 9cm seeded plates (3 P0/plate). These P0 animals were kept at 15°C and transferred to new plates twice a day, until they stopped laying eggs. The F1 animals were grown at 20°C and allowed to lay eggs for about 12h before they were counted to estimate the number of haploid genomes screened and removed. Adult F2 animals were screened for candidates with severely defective D-type motor neurons under a dissection fluorescence microscope.

Mutants (generated in the Bristol N2 background) were crossed to animals of the Hawaii (CB4856) background and the mutations mapped using fragment length polymorphism (FLP) mapping as described by 42. Following FLP mapping, we used three distinct strategies to identify candidate genes. In some cases, after sampling about 200-300 recombinants, interval sizes could be narrowed to containing 100-300 genes, which were subsequently treated with RNAi by feeding to identify possible candidate genes. Alternatively, mutations were mapped to intervals of roughly 1Mb in size and then subjected to array CGH to find the mutation as described by 43,44. As a third means to find mutations, we made use of the whole genome sequencing strategy described by 45. FLP mapping was needed to map candidates to roughly 4Mb intervals. Genomic DNA was sequenced on an Illumina sequencer and the sequencing reads aligned to the N2 reference sequence with the MAQGene software [46]. The sequencing data can be accessed via the following URLs: For strain WS3835 containing op*462*: http://www.ebi.ac.uk/ena/data/view/PRJEB4267; for strain WS3855 containing op*472*: http://www.ebi.ac.uk/ena/data/view/PRJEB4268; for strain WS4018 containing op*476*: http://www.ebi.ac.uk/ena/data/view/PRJEB4269.

### RNAi

All RNAi experiments were done in strains carrying the *eri-1*(*mg366*) mutation to render worms sensitive to RNAi [37]. For detection of candidate genes from the screen the strains subjected to RNAi additionally carried the background mutation used during the screen (either *sdn-1*(*zh20*) or *hse-5*(*tm472*)) plus the *oxIs12* transgene. For all other RNAi experiments the strain *eri-1*(*mg366*)*; sdn-1*(*zh20*) *oxIs12* was used. RNAi bacteria were seeded on nematode growth medium (NGM) agarose plates containing 1mM Ampicilin and 2mM IPTG. Worms were kept on RNAi bacteria for at least two generations before D-type motor axon guidance phenotypes were assessed.

### Scoring of D-type axon guidance defects

The D-type motor neurons were visualized with the *oxIs12*[*unc-47::gfp; lin-15(+*)]*, oxIs268*[*unc-47::gfp*] or *juIs76*[*unc-25::gfp; lin-15(+*)] transgenes. Two different decision points during axon outgrowth were scored in L1 animals: (i) the number of commissural axons turning away from the VNC (commissures/L1) and (ii) the number of commissural axons reaching the DNC (commissures reaching dorsal nerve cord). Fifty animals were scored for every genotype. Differences between pairs of strains regarding the number of commissures reaching the DNC were tested for statistical significance with a Mann-Whitney U test for non-parametric data using the freely available software R (R core team 2013) [47]. Double mutants were always compared to the corresponding single mutant with the stronger defect.

### Detection of integration site of *oxIs12*

The WGS data from all the three sequenced strains containing *oxIs12* (originally sequenced for mutation detection) were pooled and aligned to the *C. elegans* reference sequence using the CLC Genomic Workbench (www.clcbio.com) with standard alignment settings given by CLC. All the aligned reads were discarded. The non-aligned reads were aligned a second time to the *C. elegans* reference sequence, this time allowing for up to 56 misaligned bases per 76 base read. Therefore the vast majority of the reference genome remained uncovered but reads that spanned the break points of the transgene and remained unaligned after the first alignment because they contained mainly transgene sequence were aligned at the break points in this second round. Manual inspection of an interval of +/- 1cM from *sdn-1*, where we expected the integration site based on recombination frequencies, revealed two accumulations of reads with sharp break points. A blast alignment of the up to 50 non-aligned bases of the sequencing reads revealed good similarities to plasmid backbone sequences that would clearly not be found in wild type *C. elegans*.

### Determination of transgene size from WGS data

During the first alignment of the sequencing reads, all the reads originating from the transgene with endogenous counterparts will align at that endogenous position. Therefore analyzing the coverage in these areas should allow us to estimate the number of copies of the corresponding fragment present in the transgene. Figure S1 shows the coverage across the *lin-15AB* locus. While the average coverage on chromosome X (i.e. where *lin-15AB* are located) is 76.23x, it is dramatically increased at the *lin-15AB* locus to 11’030x, suggesting the presence of about 140 copies of the *lin*-15*AB*(+) construct. The part of the *lin-15AB* locus contained in the transgene (10’970bp) can be determined precisely since coverage increases from around 50x to over 4’000x at the first base included in the transgene. Using the same estimation procedure, there are roughly 170 copies of *unc-47* spanning 5180bp plus 130 copies of the *unc-54* 3’ UTR with a length of 411bp. The fact that we find 170 copies of *unc-47* and only 130 copies of the *unc-54* 3’ UTR, which are both a part of the *unc-47::gfp* construct, reflects the highly variable coverage also seen at the *lin-15AB* locus (Figure S1) and strongly suggests that the majority of the copies are neither complete nor functional. Additionally, we estimate the presence of about 150 copies of *gfp* sequence with a length of 867bp plus about 310 plasmid backbone copies of ^≈^3’000bp. Taken together this suggests that *oxIs12* has a total size of about 3.6Mb.

### qPCR for transgene size estimations

Genomic DNA was prepared form mixed stage worms of either N2 wild type, *oxIs12* or *pkIs296* animals. In *oxIs12* animals *lin-15AB* and *unc-47* were tested and in *pkIs296 dpy-20* and *gsa-1*. The following primers were used:

*lin-15AB*: fw: CCGAGAAGTGAGGAACGTTT
rv: ATGGCTTCGGCAAATTAGGT
*unc-47*: fw: ATTGGCTCTGATGCTTCTCC
rv: AGCAACCACAAAAAGACGAC
*dpy-20*: fw: TAGAGGCCATGTCTGAGGAT
rv: GGAATGCAACAAGACCCAAC
*gsa-1*: fw: CCCTGGCCCTATTCAAAAAC
rv: CGCTTGGCCTTGATTTTCTC

The average cycle number difference (Δct) of three independent experiments between N2 and the transgenic strains was determined and the copy numbers of the different constructs calculated as 2^Δct^.

In the case of *oxIs12* this approach led to the detection of about 120 copies of *lin-15AB* and 130 copies of *unc-47*, leading to a size estimation for *oxIs12* of about 2.9Mb. For *pkIs296*, qPCR indicated the presence of 14 copies of the *gsa-1* construct, which is about 9.5kb long and 65 copies of *dpy-20* (about 9.1kb) leading to a size estimation of 0.72Mb. Additionally, *pkIs296* contains an unknown amount of empty vectors [41], most likely leading to an increase in size by about two to three folds indicating that *pkIs296* is probably slightly smaller in size compared to *oxIs12.*

## Results

### A network of HSPG core proteins and HS modifying enzymes controls guidance of D-type motor neurons

**Figure 1:**
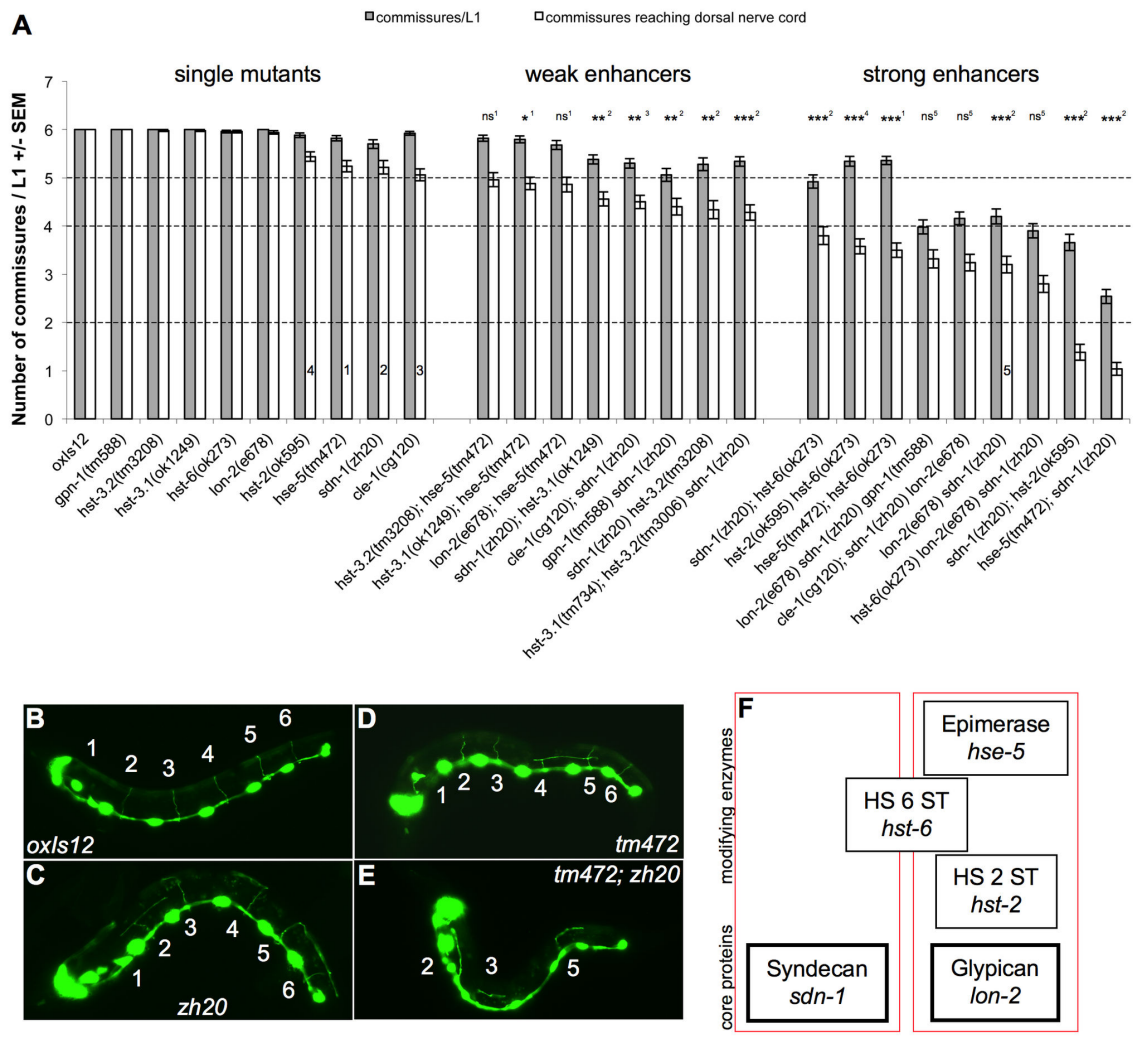
An HSPG network influences D-type motor axon guidance. A: Summary of relevant single and double mutants. All the single mutants have no or only minor defects, meaning that on average no more than one commissural axon fails to reach the dorsal nerve cord (DNC). The dashed line at 5 commissures per animal indicates the limit separating the single mutants from the weak enhancers. Weak enhancers (one to two commissural axons fail to reach the DNC, limit indicated by dashed line at 4 commissures per animal) are double mutants that are considered not to play an important role in D-type axon guidance. Strong enhancers have very clear D-type axon guidance defects (the dashed line at 2 commissures per animal is to allow for distant bars to be visually comparable). Statistical test results are indicated as follows: ns = not significant, * = p<0.05, ** = p<0.005, *** = p<0.0005. Superscripts (also shown in the bars of the corresponding strains) indicate to which strain the comparison was made: 1: *hse-5*(tm472), 2: *sdn-1*(zh20), 3: *cle-1*(cg120), 4: *hst-2*(ok595), 5: *lon-2*(e678) *sdn-1*(zh20). B-E: Representative pictures of wild type (B), *sdn-1*(zh20) (C) and *hse-5*(tm472) (D) single mutants and the strongest enhancer *hse-5*(tm472)*; sdn-1*(zh20) (E). The transgene *oxIs12* is present in all backgrounds. F: HSPG core proteins and HS modifying enzymes can be placed into two groups based on the strength of the D-type motor axon guidance defects observed in double mutants. Only the double mutants of the group of strong enhancers are considered for this network. For a complete set of strains see Figure S2. Grey bars in A represent the number of commissural axons growing away from the ventral nerve cord (VNC); white bars indicate the number of commissural axons reaching the DNC. Numbers are from 50 L1 animals +/- SEM.

During the study of the function of single HSPG core proteins and modifying enzymes in *C. elegans* it has been noted that single mutants, depending on the context, displayed specific defects in axon guidance as well as cell migration [5,16]. However, overall the defects found in these mutants were relatively weak, in particular in the guidance of D-type motor neurons (for representative pictures see Figure 1B-E). We hypothesized that the weak defects in axon guidance might be due to the presence of multiple core proteins acting in parallel. To test this, we systematically analyzed D-type motor axon guidance in double and triple mutants of the HSPG core proteins *sdn-1/Syndecan, lon-2/Glypican, gpn-1/Glypican, cle-1/Collagen XVIII* and *unc-52/Perlecan*. We observed a significant increase in guidance defects in *lon-2*(*e678*) *sdn-1*(*zh20*) double mutants, suggesting that SDN-1 and LON-2 act in parallel genetic pathways to promote D-type motor axon guidance (Figure 1A and S2). In contrast, we found no role in this process for GPN-1, the second Glypican homolog of *C. elegans*, which is in agreement with our prior observations [5]. Although the *cle-1*(*cg120*) single mutant had a weak D-type axon guidance defect, the loss of *cle-1* failed to significantly enhance either *sdn-1* or *lon-2* mutants, suggesting an indirect effect of the loss of CLE-1 function on D-type axon guidance (Figure S2). It should be noted that while *cle-1*(*cg120*) is a strong loss of function mutation it is not a null allele [48]. Residual *cle-1* function could therefore interfere with our double/triple mutant analysis. UNC-52/Perlecan has been described to play a role in Netrin signaling during ventral to dorsal migration of the distal tip cell of the *C. elegans* gonad [49]. However, our analysis of different *unc-52* alleles indicated that *unc-52* has no function in guidance of D-type motor neurons (*unc-52*(*e444*): Figure S2, data for alleles *e669, e1421*, and *gk3* not shown). Since *unc-52* null alleles are lethal only hypomorphic alleles were used. We also tested mutations in *agr-1/Agrin* and *VC5.2*, a gene with similarity to Perlecan but found that these core proteins are not involved in D-type motor axon guidance (data not shown).

Earlier work indicated the importance of cell type-specific modifications of the HS chains by various HS modifying enzymes [17]. Different modifications can confer distinct function to a common HSPG core protein, which led to the proposal of an HS code for HSPGs [16]. We therefore built double and triple mutants of genes coding for a core protein and one of the HS sulfotransferases *hst-2, hst-3.1, hst-3.2, hst-6* or the C5-epimerase *hse-5* with the aim to find the HS code for SDN-1 and LON-2 for their function in dorsal guidance of D-type motor neurons. Mutations in *hst-2* and *hse-5* strongly enhanced D-type axon guidance defects of the *sdn-1*(*zh20*) mutant but resulted in no enhancement when combined with *lon-2*(*e678*) (Figure 1A and S2). This suggests that HST-2 and HSE-5 might act mainly on LON-2. This is compatible with the finding that *lon-2, hst-2*, and *hse-5* are expressed in the hypodermis [16,50,51] while *sdn-1* and *hst-6* expression is found mainly in neurons [5,16].

There are two *C. elegans* genes coding for a protein with potential HS-3-O sulfotransferase activity, *hst-3.1* and *hst-3.2*. The *hst-3.1; hst-3.2 sdn-1* as well as the *hst-3.1; hst-3.2 lon-2* triple mutants failed to further increase D-type motor axon guidance defects, indicating that HS-3-O sulfotransferase activity is not important for this axon guidance event (Figure 1A and S2).

A particular situation was found with *hst-6*. A mutation in this gene clearly enhances *sdn-1*(*zh20*) while the *lon-2*(*e678*) *hst-6*(*ok273*) double mutant shows little defect (Figure S2), indicating that HST-6 acts on LON-2 and in parallel to SDN-1. However, both the *hst-6 hst-2* and *hse-5; hst-6* double mutants do show increased defects (Figure 1A) indicating that HST-6, besides acting on LON-2, modifies the HS chains of an additional core protein. Because *hst-6*(*ok273*) does not further enhance the *lon-2 sdn-1* double mutant (Figure 1A), it is likely that this additional HSPG core protein is in fact SDN-1, rather than another uncharacterized HSPG. Taken together, our data show that SDN-1 and LON-2 are the two HSPG core proteins relevant for D-type motor axon guidance. We were also able to determine the HS code of the sugar side chains of these two core proteins: HSE-5 and HST-2 act mainly on LON-2 in D-type dorsal guidance, whereas modification by HST-6 is required both on SDN-1 and LON-2. We propose that the resulting modified HS chains on LON-2 and SDN-1 have the affinity to bind to certain as yet unknown axon guidance molecules.

### A screen for genes acting upstream or downstream of HSPGs during D-type motor axon guidance

From our mutational analysis of HSPG core proteins and HS modifying enzymes we concluded that HSPGs are involved in at least two pathways influencing D-type motor axon guidance, one involving SDN-1 the second one HSE-5 and HST-2, acting on LON-2. We decided to use this system in a forward genetic enhancer screen in order to identify additional genes acting in these two pathways (Figure S3). Of all the tested double mutant combinations, the *hse-5*(*tm472*)*; sdn-1*(*zh20*) animals displayed the most severe defects. Therefore we separately mutagenized *sdn-1*(*zh20*) *oxIs12* and *hse-5*(*tm472*)*; oxIs12* animals and screened the F2 generation for candidates phenocopying the *hse-5; sdn-1* double mutant phenotype. The transgene *oxIs12* was used to label the D-type motor neurons with *gfp*. We screened approximately 11’000 genomes for each of the two approaches. Isolated candidates were mapped using fragment length polymorphisms (FLPs) as described by 42 either until the phenotypically relevant mutation was found or until intervals were small enough to use arrayCGH [44] or whole genome sequencing [45] for detection of the mutation. We isolated a total of 14 candidates from the two screens and for all but four of these the relevant mutation could be identified (Table 1). Many of the genes detected are related to the Netrin pathway (e.g. *unc-5, unc-130, max-1*), consistent with the fact that UNC-6/Netrin is the major axon guidance cue responsible for circumferential guidance of D-type motor axons [19].

**Table 1:** Summary of *sdn-1*(*zh20*) and *hse-5*(*tm472*) enhancers.

Allele	Gene	Amino acid change	Genomic environment
*op459*	*unc-130*	R219K	CAATGGATCGTTTCTGCGCA**A**AAGGAAGAGATACAAGAAAA
*op460*	*unc-5*	C25Y	TAAGCCATTGCGTCTTCAAT**A**CCGAGCCAATCACGCAACAA
*op462*	*zag-1*	Q485stop	AGGGTCTCTTCTCGTGCGAT**T**AATGTGACAAGGTGTTCGGC
*op464*	*unc-73*	intron	TATTTGAAGTTACTTCGTAA**A**TTTTTTATTTGACTGAAATG
*op468*	*unc-5*	S559stop^a^	5’ flanking region: ACCTCACCTCAAACCAATCG
			3’ flanking region: AGAGTAATTGTCTATCAAAA
*op469*	*unc-5*	G254E	TCCAGCTCCATTGAACGACG**A**AGAGTACTGTAAGGGAGAAG
*op472*	nd	-	-
*op474*	nd	-	-
*op476*	nd	-	-
*op477*	*unc-5*	G259R	ACGACGGAGAGTACTGTAAG**A**GAGAAGAAGAAATGACGAGA
*op479*	*unc-53*	R1258stop	TTACTTCCTCAACTACAATC**T**GAATGTTCATGCACGGTGCC
*op480*	nd	-	-
*op481*	*zfp-1*	W601stop	ACAGCTGTTGGAACGACAAT**A**GGATCAAGGATCAAGTCTTC
*op482*	*max-1*	W878stop	CTTCGAATGAGACATTATTG**A**GGCCATTTGGCACTGACAGA

a op*468* is a deletion of 337bp leading to a frame shift, E555 being the last correct amino acid, introducing a stop codon at position 559. All other mutations are G to A point mutations; the mutated base is in bold and underlined

**Figure 2:**
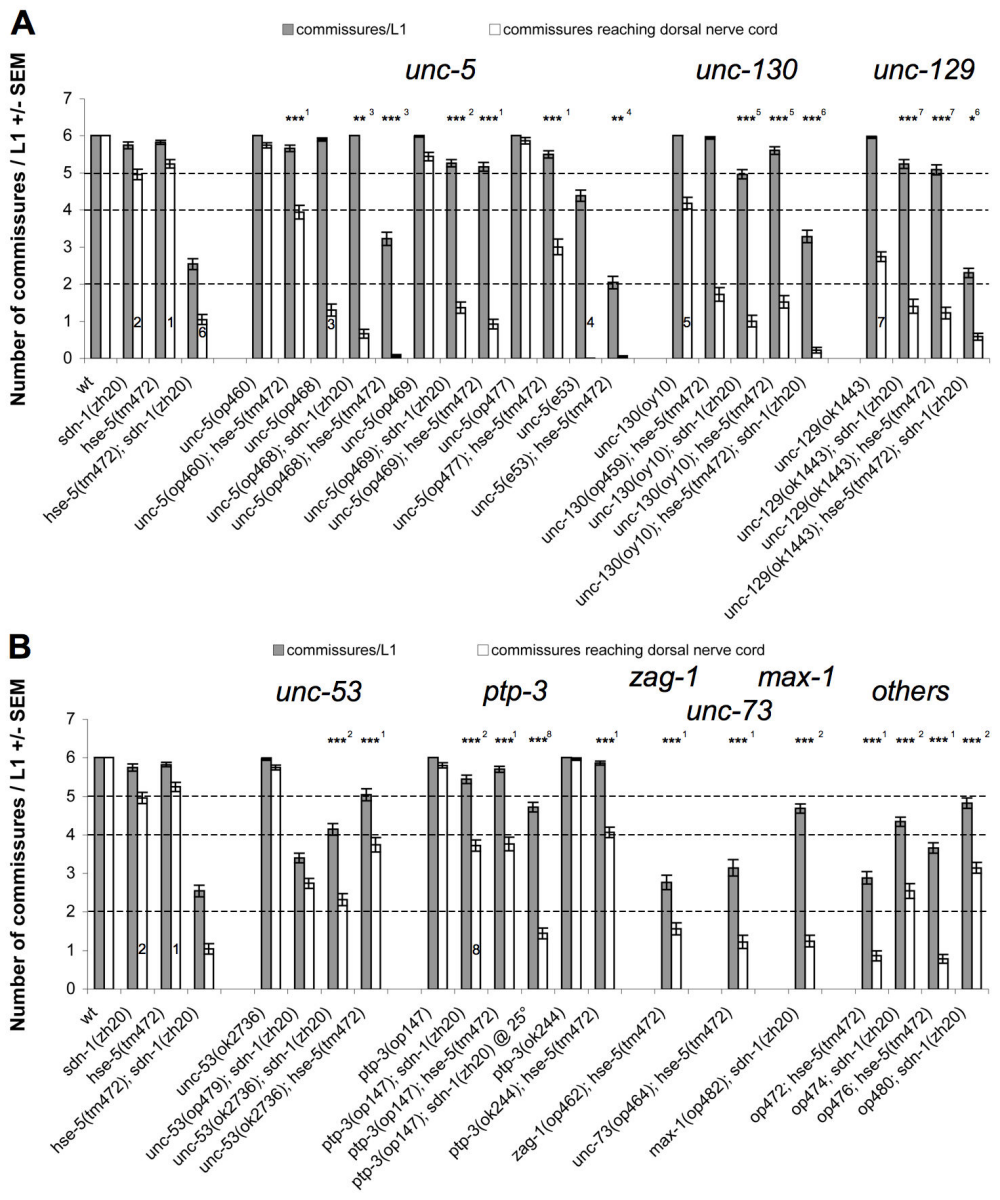
Axon guidance defects of *sdn-1* and *hse-5* enhancers. A: Netrin pathway mutants, as single mutants and in combination with *hse-5*(tm472) and *sdn-1*(zh20). Of the *unc-5* alleles only op*468* has a single mutant phenotype, but is still weaker than the *e53* reference allele. Mutations in *unc-129* and *unc-130* show similar defects, both as single mutants as well as in double mutants with *sdn-1*(zh20) or *hse-5*(tm472). B: Mutations in *unc-53* and *ptp-3* enhance both *sdn-1* and *hse-5* mutations. op*147* shows a strong temperature sensitive phenotype and is considered a strong allele at 25°. *ok244* on the other hand shows a weaker phenotype. We also isolated alleles in *max-1*, *zag-1*, and *unc-73*. op*472* and op*476* map to the same interval on chromosome I but whole genome sequencing failed to find a mutation in either strain. op*474* and op*480* were difficult to map and therefore not characterized further. Grey bars represent the number of commissural axons growing away from the VNC; white bars indicate the number of commissural axons reaching the DNC. Dashed lines indicate limits according to Figure 1A. Numbers are from 50 L1 animals +/- SEM. Statistical test results are indicated as follows: ns = not significant, * = p<0.05, ** = p<0.005, *** = p<0.0005. Superscripts (also shown in the bars of the corresponding strains) indicate to which strain the comparison was made: 1: *hse-5*(tm472), 2: *sdn-1*(zh20), 3: *unc-5*(op468), 4: *unc-5*(e53), 5: *unc-130*(oy10), 6: *hse-5*(tm472)*; sdn-1*(zh20), 7: *unc-129*(ok1443), 8: *ptp-3*(op147)*; sdn-1*(zh20).

Of the four *unc-5* alleles recovered in the screen, only op*468* has clear D-type axon guidance defects as a single mutant (Figure 2A). The op*468* allele carries a deletion of 337bp resulting in a frame shift and a premature termination of translation. Nevertheless, op*468* seems to be a hypomorphic allele judged by the fact that the reference allele *unc-5*(*e53*) showed more pronounced defects, both on its own and in combination with *hse-5*(*tm472*) (Figure 2A). The other *unc-5* mutations op*460*, op*469*, and op*477* are very weak missense point mutations affecting the extra cellular domain indicating that the amino acid sequence of UNC-5 is highly sensitive to changes in the HSPG sensitized background, hinting at a possible interaction between UNC-5 and HS chains on SDN-1 or LON-2 or both.

The TGFβ ligand UNC-129 and its transcription factor UNC-130 have been described to act in circumferential axon guidance [33] and participate in Netrin signaling [24]. The *unc-130* allele recovered from our screen affects the neighboring amino acid mutated in the *oy10* reference allele within the conserved forkhead domain. Mutations in *unc-129* and *unc-130* lead to comparable defects in D-type motor axon guidance (Figure 2A). With *max-1* we also found a gene that is thought to act downstream of *unc-6*/Netrin [34].

We also identified mutations in a number of genes whose link to the Netrin pathway is less clear. UNC-53, like its three human homologs the Navigators NAV1-3, is important for cytoskeletal rearrangements to control cell migration and axon guidance (reviewed in 52). UNC-53 is known to play a role in longitudinal axon guidance and cell migration in *C. elegans* [53]. We found that both the allele from our screen, *unc-53*(*op479*), which carries a nonsense mutation and the deletion allele *unc-53*(*ok2736*) clearly enhance the *sdn-1*(*zh20*) mutation (Figure 2B), demonstrating that UNC-53 also influences circumferential axon guidance events.

During our search for the gene affected by the op*479* mutation, we found that the RNAi clone for *ptp-3* gave a positive phenotype. While sequencing revealed that there was no mutation in *ptp-3* in candidate op*479*, the Tc1 transposon insertion allele *ptp-3*(*op147*) isolated earlier in our lab led to a clear increase of D-type motor axon guidance defects in the *hse-5* or *sdn-1* mutant background. It has been reported that op*147* is temperature-sensitive [31] and indeed growing *ptp-3*(*op147*)*; sdn-1*(*zh20*) animals at 25° increases their defects while *ptp-3*(*op147*)*; hse-5*(*tm472*) animals do not survive at 25° (Figure 2B). PTP-3 is a receptor tyrosine phosphatase of the LAR (Leukocyte Common Antigen related) type. Its homolog in *Drosophila* has been shown to interact with HS chains on Syndecan and Dallylike/Glypican during the process of synapse formation [54,55]. In order to explore the relevance of LAR signaling during D-type axon guidance in more detail, we tested other LAR signaling components. NID-1/Nidogen is an extracellular binding partner of LAR and UNC-10/RIM binds LAR intracellularly (for a review, see 56). However, neither *nid-1*(*cg119*) nor *unc-10*(*md1117*) enhanced *sdn-1*(*zh20*) or *hse-5*(*tm472*) (data not shown), indicating that while PTP-3/LAR influences D-type motor axon guidance this action is not going through standard LAR signaling.

The op*462* mutation introduces a premature stop codon in *zag-1*, a gene coding for a Zn finger and homeodomain transcription factor that controls differentiation and axon guidance of multiple neuronal cell types, including the D-type motor neurons, likely by regulating the expression of as yet unknown transcriptional target genes [57]. We also identified in op464 a mutation in an intron of *unc-73*, a guanine nucleotide exchange factor known to affect multiple axon guidance decisions [58] (Figure 2B). Whether this intronic mutation is the sequence change leading to the D-type axon guidance defects is unclear. Since it affects the 5th base in the intron it could potentially lead to a splicing defect.

**Figure 3:**
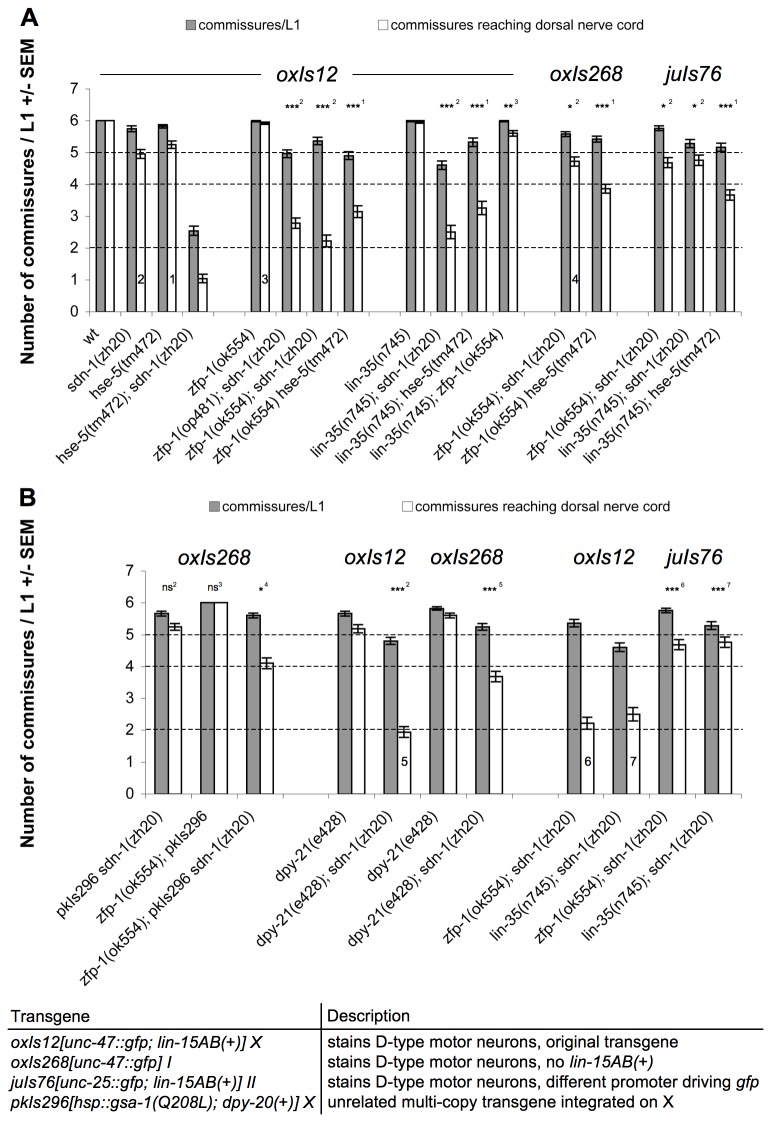
Mutations in *zfp-1* or *lin-35* enhance axon guidance defects in an *oxIs12*-dependent manner. A: The *zfp-1*(op481) point mutation, the *zfp-1*(ok554) deletion and *lin-35*(n745) lead to a very similar enhancement of defects when combined with *sdn-1*(zh20) or *hse-5*(tm472). However *zfp-1*(ok554) and *lin-35*(n745) are not enhancing each other. The defects of *zfp-1*(ok554)*; sdn-1*(zh20) and *lin-35*(n745)*; sdn-1*(zh20) disappear if *oxIs12* is replaced by either *oxIs268* or *juIs76*. B: Attempts to reconstitute the defects. (i) *pkIs296* enlarges the X-chromosome of *oxIs268; zfp-1*(ok554)*; sdn-1*(zh20) animals leading to a slight increase in defects, however not to the level of *zfp-1*(ok554)*; sdn-1*(zh20) *oxIs12* animals (ii). The *dpy-21*(e428) mutation behaves the same as *zfp-1* or *lin-35* mutations. The *dpy-21*(e428)*; sdn-1*(zh20) double mutant has severe D-type axon guidance defects if *oxIs12* is present but not if *oxIs268* is used to stain the D-type motor neurons. The phenotypes of *zfp-1*(ok554)*; sdn-1*(zh20) and *lin-35*(n745)*; sdn-1*(zh20) with either *oxIs12* or *juIs76* are shown as comparison. Grey bars represent the number of commissural axons growing away from the VNC; white bars indicate the number of commissural axons reaching the DNC. Dashed lines indicate limits according to Figure 1A. Numbers are from 50 L1 animals +/- SEM. Statistical test results are indicated as follows: ns = not significant, * = p<0.05, ** = p<0.005, *** = p<0.0005. Superscripts (also shown in the bars of the corresponding strains) indicate to which strain the comparison was made: 1: *hse-5*(tm472), 2: *sdn-1*(zh20), 3: *zfp-1*(ok554), 4: *zfp-1*(ok554)*; sdn-1*(zh20)*; oxIs268*, 5: *dpy-21*(e428)*; sdn-1*(zh20) *oxIs12*, 6: *zfp-1*(ok554)*; sdn-1*(zh20) *oxIs12*, 7: *lin-35*(n745)*; sdn-1*(zh20) *oxIs12*. The table summarizes the different transgenes used, indicates their composition and the chromosome in which they are integrated.

Furthermore, we found a premature stop codon in the gene *zfp-1* (Figure 3). ZFP-1 has not previously been described to act in UNC-6/Netrin signaling or HSPG related signaling. We therefore decided to focus our attention on this gene in order to understand how it influences HSPG dependent Netrin signaling.

### Axon guidance defects of *zfp-1* and *lin-35* mutants depend on the presence of the *oxIs12* transgene

ZFP-1 is a zinc finger protein that has been found to control *C. elegans* vulval development by counteracting a large group of genes called synthetic multivulva (SynMuv) genes [36]. SynMuv genes can be separated into three classes, SynMuvA/B/C. While single mutants of any SynMuv gene show no aberrant vulva development, double mutants of two genes belonging to different SynMuv classes will result in the typical multivulval phenotype. ZFP-1 has also been suggested to act in the RNAi pathway, again counteracting SynMuv genes [59,60]. The human homolog of *zfp-1* is called AF10 and is involved in pediatric acute myeloid leukemia (AML) [61] making *zfp-1* an interesting gene to explore.

The op*481* mutation introduces a premature stop codon in the ZFP-1 open reading frame (Table 1). A second *zfp-1* allele, *ok554*, deletes part of the gene and shows an identical axon guidance phenotype as op*481* (Figure 3A).

During our efforts to create an RNAi-sensitive strain containing *sdn-1*(*zh20*), we found that the double mutant *lin-35*(*n745*)*; sdn-1*(*zh20*) displayed D-type axon guidance defects even without RNAi (Figure 3A). *lin-35*, a member of the SynMuv B class of genes, codes for the *C. elegans* homolog of the human Retinoblastoma tumor suppressor protein (pRb) (for a comprehensive review of pRb function in cancer and development see 62). The diversity of functions of pRb is a result of the ability of pRb to interact with more than 150 different proteins and even act as an adaptor by interacting with more than one protein at a time [63].

Since *zfp-1* is a SynMuv suppressor gene and *lin-35* a SynMuv gene we wondered whether other SynMuv pathway genes would influence D-type axon guidance in a similar way. To test this we knocked down all the known SynMuv genes [64] and SynMuv suppressors [36] by RNAi. However, none of the genes tested resulted in a similar phenotype as *zfp-1* or *lin-35* (Table S1). In further attempts we explored the influence of Argonaute genes as well as genes involved in chromatin remodeling, but again failed to find any strong enhancers (Table S1).

The *oxIs12* D-type reporter transgene we used for our screen and all subsequent analyses contains multiple copies of the genes *lin-15A* and *lin-15B* as a co-injection marker. Both genes are part of the same operon known as the *lin-15AB* locus. Mutations affecting both genes together result in a strong SynMuv phenotype. Since *lin-15AB* are SynMuv genes themselves, we developed concerns regarding the use of a transgene such as *oxIs12* that will lead to the overexpression of *lin-15AB*. To address this issue, we tested the phenotype of *zfp-1*(*ok554*)*; sdn-1*(*zh20*) and *zfp-1*(*ok554*) *hse-5*(*tm472*) animals using the transgene *oxIs268*, which contains the same *gfp* reporter (*unc-47::gfp*) as *oxIs12* but without any co-injection marker. Strikingly, in the *oxIs268* background, *zfp-1*(*ok554*) completely failed to enhance the defects of *sdn-1*(*zh20*) mutants and only weakly enhanced the axon guidance defects of *hse-5*(*tm472*) mutants (Figure 3A). Further experiments however suggested that *lin-15AB* overexpression in *oxIs12* was not the cause for the enhancing effect of this transgene (data not shown).

Our findings prompted us to ask whether *oxIs12* would generally influence D-type axon guidance and tested other mutations from our screen with different transgenes. Only one other gene, namely *lin-35*, showed an *oxIs12* transgene dependence similar to *zfp-1* (Figure 3A). It is important to note that none of the major genes involved in D-type axon guidance such as *unc-5, unc-6, unc-40, unc-129, unc-130* as well as none of the genes found in our genetic screen or candidate gene approach showed axon guidance phenotypes that depended on the *oxIs12* transgene (data not shown), except *zfp-1* and *lin-35*. Taken together, our data clearly indicate that the axon guidance defects of *zfp-1*(*ok554*)*; sdn-1*(*zh20*) and *lin-35*(*n745*)*; sdn-1*(*zh20*) animals strongly depend on the presence of the *oxIs12* transgene and that the over-expression of *lin-15AB* from *oxIs12* is most likely not relevant for this effect.

### Molecular characterization of *oxIs12*

**Figure 4:**
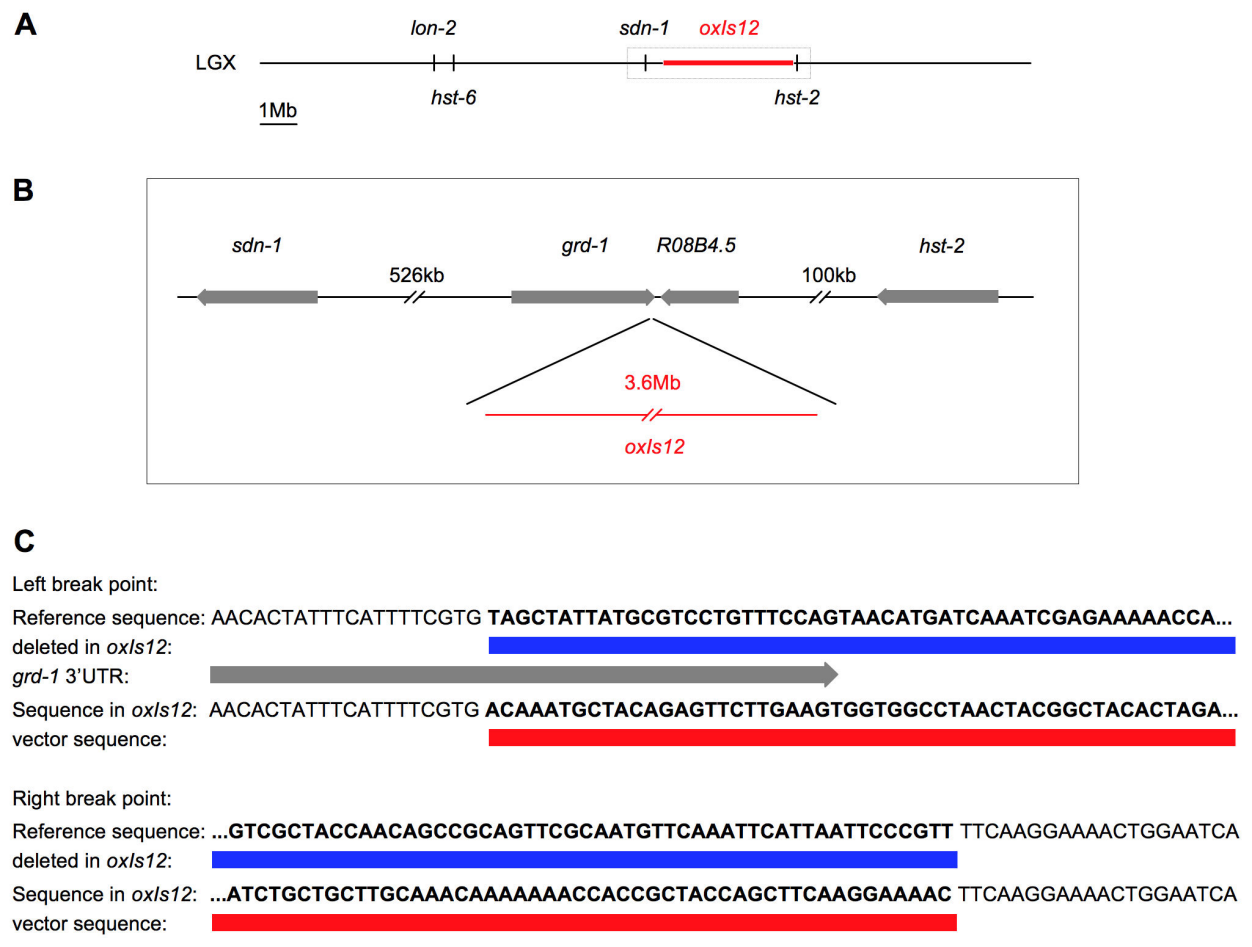
Molecular characterization of *oxIs12*. A: X chromosome with the integrated transgene *oxIs12*, drawn to scale. *oxIs12* is approximately 3.6Mb in size and thus substantially enlarges the X chromosome. B: Enlargement of the boxed region in A. Whole genome sequencing data place *oxIs12* within the 3’ UTR region of *grd-1*, close to *R08B4.5*. C: Break points introduced by *oxIs12*. The insertion introduced a deletion of 232bp (blue boxes) including the last 25bp of the 3’ UTR of *grd-1* (grey arrow) and a large part of the intergenic region between *grd-1* and *R08B4.5*. The sequencing reads spanning the break points also reveal a small part of the transgene sequence (red boxes). In both cases a BLAST search indicates the presence of vector backbone sequence, which would usually not be found in the *C*. *elegans* genome.

Transgene-specific effects could occur due to genetic or epigenetic changes introduced by the transgene at its integration site. In order to test this possibility we sought to determine the precise site of *oxIs12* integration. From previous genetic analysis, we expected *oxIs12* to be less than 1cM away from *sdn-1* on the X chromosome (Figure 4A). Analysis of whole genome sequencing data of animals containing *oxIs12* led to the identification of two locations with clear break points that are 232bp apart and represent a short deletion introduced by the integration of *oxIs12*, which is located in the 3’ UTR of *grd-1* and close to *R08B4.5* (Figure 4B, C). Unfortunately we were unable to test the impact of *grd-1* or *R08B4.5* in axon guidance with a mutation. However, RNAi against *grd-1* and *R08B4.5* failed to influence D-type motor axon guidance. Taken together, our observations support the conclusion that the transgene-specific effect of *oxIs12* is not due to disruption of a gene at the site of integration.

In order to gain insight into the size of *oxIs12*, we further analyzed the whole genome sequencing data (see Materials and Methods). This suggested that *oxIs12* has a total size of about 3.6Mb, increasing the size of the X chromosome by approximately 20%. Additionally, we performed qPCR for *lin-15AB* and *unc-47* in *oxIs12* animals and compared it to the N2 wild type. This approach led to a size estimation for *oxIs12* of about 2.9Mb. The fact that the qPCR data led to a lower estimation than the whole genome sequencing data can probably be explained by the fact that different parts of one construct are contained in different copy numbers.

Given the large size of *oxIs12* we hypothesized that the transgene dependence seen in *zfp-1*(*ok554*)*; sdn-1*(*zh20*) animals could be a result of the increased size of the X chromosome due to the integration of the multi-copy transgene *oxIs12* on X. We therefore crossed *pkIs296*[*gsa-1(Q208L*)*; dpy-20(+*)], another unrelated multi-copy transgene integrated on X, into *oxIs268; zfp-1*(*ok554*)*; sdn-1*(*zh20*) animals (*oxIs268* is integrated on chromosome I). qPCR for the components of *pkIs296* indicated that *pkIs296* is probably slightly smaller in size compared to *oxIs12*. Indeed, *oxIs268; zfp-1*(*ok554*)*; sdn-1*(*zh20*) *pkIs296* animals showed increased defects compared to *oxIs268; zfp-1*(*ok554*)*; sdn-1*(*zh20*), although not to the level of defects seen in *zfp-1*(*ok554*)*; sdn-1*(*zh20*) *oxIs12* (Figure 3B).

Increasing the size of the X chromosome could interfere with dosage compensation. If the dosage compensation complex (DCC) has to cover a bigger chromosome, this could lead to a reduction of DCC density and consequently to an increased transcription of X-linked genes. Indeed, many of the genes with a link to D-type motor axon guidance and HSPGs are located on the X chromosome, e.g. *unc-6, lon-2, sdn-1, hst-2* and *hst-6*. If loss of *zfp-1* or *lin-35* function were to reduce dosage compensation efficiency, then a mutation in the gene *dpy-21*, a member of the DCC [38], might lead to similar axon guidance defects in our specific setup. Indeed, the *dpy-21*(*e428*)*; sdn-1*(*zh20*) *oxIs12* strain had increased D-type motor axon guidance defects compared to the *oxIs268; dpy-21*(*e428*)*; sdn-1*(*zh20*) strain (Figure 3B). This finding clearly shows that the integration of a multi-copy transgene on the X chromosome in the sensitive genotype used in our mutagenesis screen to find novel HSPG related axon guidance genes has led to the discovery of two genes that influence axon guidance in an indirect way.

## Discussion

### The HSPG network of D-type motor axon guidance

Our efforts to shed light on the role played by HSPGs and HS modifying enzymes during guidance of D-type motor axons clearly showed that SDN-1/Syndecan and LON-2/Glypican have parallel functions in this process. Other core proteins such as GPN-1/Glypican, CLE-1/Collagen XVII, UNC-52/Perlecan or AGR-1/Agrin did not seem to be involved. Minor defects could however be observed, which might be the result of pleiotropic defects, since it is very likely that each core protein has a wide range of functions.

Our analysis of the modifying enzymes revealed that mutations in *hst-2, hse-5*, and *hst-6* clearly enhance *sdn-1*(*zh20*) but not *lon-2*(*e678*), indicating that they all modify HS chains of LON-2, and that they either do not modify SDN-1, or that these modifications are not important for SDN-1 function in D-type axon guidance. HS-3-O sulfotransferase activity coming from *hst-3.1* or *hst-3.2* does not seem to be important for D-type motor axon guidance, at least under the conditions and in the genotypes tested. Surprisingly, we found that *hst-6 hst-2* and *hse-5; hst-6* double mutants, but not *hse-5; hst-2*, showed enhanced D-type motor axon guidance defects. Since the *lon-2 hst-6 sdn-1* triple mutant was indistinguishable from the *lon-2 sdn-1* double mutant, we hypothesized that HST-6 is indeed acting on the HS chains of both LON-2 and SDN-1, but that the lack of modification on SDN-1 is not strongly detrimental.

The strain with the strongest defects is the *hse-5*(*tm472*)*; sdn-1*(*zh20*) double mutant. Theoretically this phenotype should be phenocopied by mutating all the relevant core proteins. However the double mutant of *lon-2* and *sdn-1*, which were shown to act in D-type axon guidance, has less dramatic defects. This could point to the presence of a third core protein or it could be the result of a dominant negative function of HS chains on LON-2, which lack the modification introduced by HSE-5 and therefore have altered binding affinities. The second explanation could be tested in an *hse-5*(*tm472*)*; lon-2*(*e678*) *sdn-1*(*zh20*) triple mutant, which should then be phenotypically identical to the *lon-2*(*e678*) *sdn-1*(*zh20*) double mutant. Unfortunately, this triple mutant is lethal and *lon-2* RNAi in the *sdn-1*(*zh20*) *oxIs12* and *hse-5*(*tm472*)*; sdn-1*(*zh20*) *oxIs12* background had no effect on axon guidance (data not shown).

Taken together, the data indicate that there is a tight network of HSPGs and HS modifying enzymes influencing dorsal guidance of D-type motor axons. The ready availability of mutations in all HSPG core proteins and HS modifying enzymes enabled us to describe the HSPG network relevant for efficient dorsal axon guidance in the worm and establish the HS code for both involved core proteins. The HS chains of LON-2 need to be modified by HST-2, HSE-5 and HST-6 to be able to exert their function. HS chains on SDN-1 seem to require HST-6 function only.

### A screen for genes acting upstream or downstream of HSPGs during D-type motor axon guidance

In order to find new genes involved in HSPG-dependent axon guidance, we designed two approaches for a forward genetic screen. We chose to use either *sdn-1*(*zh20*) or *hse-5*(*tm472*) as sensitive backgrounds, because the double mutant of these two genes showed the most severe axon guidance defects in D-type motor axons and we assumed that the function of those two genes would be most divergent. Furthermore, using a sensitive mutant background should allow us to recover mutations that only have a weak phenotype on their own, similar to the two background mutations. For both backgrounds we screened about 11’000 haploid genomes. It became clear upon mapping that we did not saturate the genome with the mutagenesis because (i) we failed to recover the positive controls *sdn-1* and *hse-5* or some obvious candidates such as *unc-6* and *unc-40* and (ii) all the genes found to be mutated in a candidate were only recovered once, with the clear exception of *unc-5* for which we found 4 different alleles. It is certainly remarkable that *unc-5* is so prone to the accumulation of mutations in our screening set up. Interestingly, the three point mutants op*460*, op*469* and op*477* alone have little or no effect on axon guidance; a phenotype can be observed only when combined with a mutation in *sdn-1* or *hse-5*, i.e. when HSPG related signaling is impaired. There are multiple ways of interpreting this finding: a) HSPGs could bind via their HS chains to UNC-5 to facilitate signaling through it or locally concentrate a ligand, which may make the amino acid sequence of UNC-5 particularly sensitive to changes because changing the amino acid sequence could alter the HS binding efficiency. b) HSPGs could influence a signaling pathway acting in parallel to UNC-6/Netrin, such as the Slit/Robo pathway that has been shown to act in ventral axon guidance [65,66]. Altering signaling through this parallel pathway could render D-type motor axons particularly sensitive to any further changes in axon guidance signaling. It is interesting to note that the double mutant *hse-5*(*tm472*)*; unc-5*(*e53*) and the two triple mutants *hse-5*(*tm472*)*; unc-129*(*ok1443*)*; sdn-1*(*zh20*) and *unc-130*(*oy10*)*; hse-5*(*tm472*)*; sdn-1*(*zh20*) all resemble *hse-5*(*tm472*)*; sdn-1*(*zh20*) double mutants, hinting that UNC-5, HSE-5, SDN-1 and UNC-129/130 could possibly function in a common pathway.

**Figure 5:**
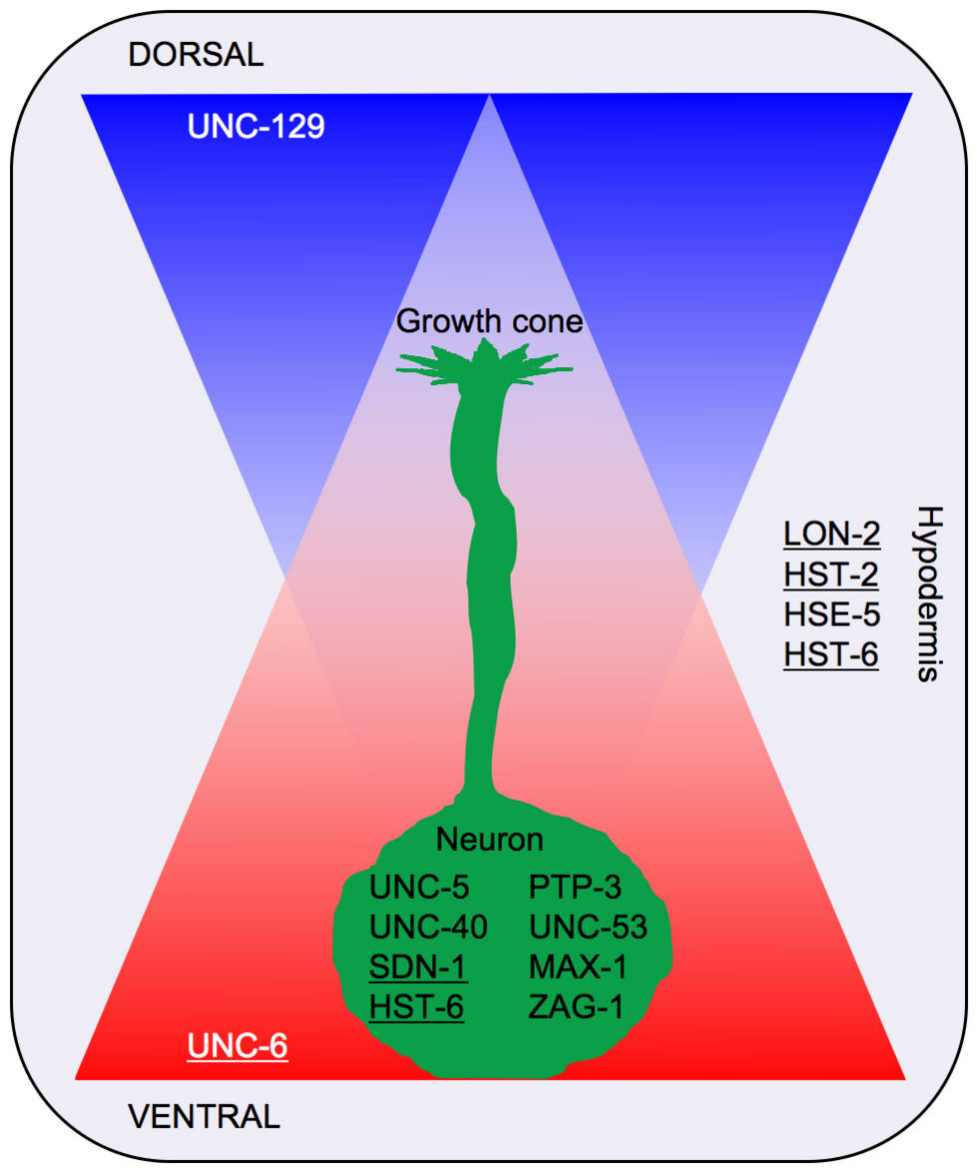
Factors involved in dorsal axon guidance of D-type motor neurons. The two UNC-5 ligands UNC-6 (red) and UNC-129 (blue) form opposing gradients with high concentrations of UNC-6 ventrally and high UNC-129 concentrations on the dorsal side of the worm. The ventrally outgrowing growth cone (green) initially uses the UNC-5 receptor to respond to high UNC-6 concentrations. The more the growth cone approaches the dorsal side, the lower the UNC-6 concentration becomes while the concentration of UNC-129 increases, which is thought to induce a switch in UNC-6 signaling in the growth cone to UNC-5 + UNC-40 signaling. This enables the growth cone to maintain its response to the repulsive UNC-6 signal [24]. The HSPG SDN-1 is expressed in the neuron [5] and requires HST-6 function to modify its HS chains. SDN-1 could, through its HS chains, influence the interaction of UNC-6 and/or UNC-129 with UNC-5. Our genetic data suggest that PTP-3 is playing a role in this process too, but it remains an open question what its precise function is. Furthermore UNC-53 is involved in cytoskeletal remodeling [52] and MAX-1 [34] and ZAG-1 [57] are transcription factors with a possible function in the neuron. The HSPG LON-2 is expressed in the hypodermis [50]. Its function could be to establish and/or maintain the gradients of UNC-6 and/or UNC-129 to provide an ideal substrate for the growth cone on its way to the dorsal side. For this function LON-2 requires the enzymes HST-2, HSE-5 and HST-6 to modify its HS chains. Underlined protein names indicate that these genes are located on the X chromosome.

The initial aim of the screen was to find genes that enhance e.g. *sdn-1*(*zh20*) and fail to enhance *hse-5*(*tm472*). Remarkably, none of the enhancers isolated in the screen were found to have this effect. Rather, all mutations isolated enhanced both *sdn-1*(*zh20*) and *hse-5*(*tm472*) to similar levels. This indicates that the function of SDN-1 and LON-2 could converge on a common pathway during guidance of D-type motor axons. The results from the screen suggest that this common pathway could be UNC-6/Netrin signaling (Figure 5). The sensitivity for mutations in *unc-5* seen in our screen could suggest that HS chains attached to LON-2 and/or SDN-1 may bind to UNC-5. Alternatively, LON-2 and/or SDN-1 could bind via their HS chains to the UNC-5 ligands UNC-6/Netrin or UNC-129/TGFβ, either to increase their local concentration or to facilitate their interaction with UNC-5. Additionally a third pathway in parallel to UNC-6/Netrin signaling could be influenced by HSPGs. Since LON-2 and SDN-1 were shown to carry distinct HS codes and are expressed in different tissues (hypodermis and neurons, respectively) they may interact differentially with UNC-6, UNC-129 or other unknown ligands. Finally, SDN-1 could also act as a receptor in its own right and activate a signal transduction cascade upon ligand binding.

The finding that the transmembrane tyrosine phosphatase PTP-3 plays a role in D-type motor axon guidance suggests a requirement for tyrosine phosphorylation in this process. *Drosophila* LAR, the homolog of PTP-3, has been shown to bind to HS chains on Syndecan and Glypican [54,55]. A similar function for PTP-3 is therefore likely. Furthermore, we describe for the first time a function in dorsal axon guidance for UNC-53, a gene involved in cytoskeleton remodeling previously shown to participate in longitudinal axon guidance (Figure 5). Whether PTP-3 and UNC-53 influence Netrin signaling or act in parallel pathways remains an open question.

### The large multi-copy transgene *oxIs12* influences D-type axon guidance in combination with a mutation in *zfp-1* or *lin-35*

In our search for novel components that influence HSPG dependent D-type motor axon guidance, we also identified two apparently unrelated genes affecting the process, *zfp-1* and *lin-35. zfp-1* has been shown to counteract SynMuv genes [36] and to have a positive influence on the RNAi pathway [59,60]. On the other hand, many SynMuv genes have a negative influence on RNAi [67], which is why *lin-35* mutants are often used as sensitive background for RNAi experiments. However, no other SynMuv or SynMuv suppressor genes showed the same phenotypes as *lin-35*(*n745*) or *zfp-1*(*ok554*), suggesting that their influence on axon guidance is not the representation of a general effect of these two classes of genes.

During our investigation of the general effect on axonal guidance of SynMuv genes, we noted that the *oxIs12*[*unc-47::gfp; lin-15AB(+*)] transgene used to visualize the D-type motor neurons was required to induce D-type motor axon guidance defects in *zfp-1*(*ok554*)*; sdn-1*(*zh20*) and *lin-35*(*n745*)*; sdn-1*(*zh20*) animals. Two other transgenes (*oxIs268*[*unc-47::gfp*] and *juIs76*[*unc-25::gfp; lin-15AB(+*)]) did not provoke major D-type axon guidance defects in those two double mutants, suggesting that neither the *gfp* construct nor the marker used in the transgenes are playing a role. *oxIs12* is not generally influencing D-type motor axon guidance. The transgene dependence phenotype is very specific for *zfp-1* and *lin-35* mutations in combination with a second mutation in an HSPG network gene.

### Multi-copy transgenes integrated on X could interfere with dosage compensation

Our analysis of *oxIs12* resulted in an estimated size of approximately 3.6Mb. This is a significant amount of additional DNA in an animal with a wild-type genome size of about 100Mb and is increasing the size of the X chromosome, which is 17.7Mb long, by approximately 20%. Since many genes contained in the HSPG network (Figure 1) (*hst-6, lon-2, sdn-1, hst-2*) as well as *unc-6/Netrin* are located on X (Figure 5) we hypothesized that the change in size of the X chromosome is what made *oxIs12* special.

*oxIs12* could disrupt dosage compensation by diluting the dosage compensation complex (DCC) across the whole enlarged X chromosome. This would lead to an upregulation of genes on the X chromosome and ultimately to D-type axon guidance defects when combined with an *sdn-1*(*zh20*) mutation, since there are many other genes on X that are relevant for proper guidance of D-type motor axons. A clear confirmation of this hypothesis came from the finding that the dosage compensation mutant *dpy-21*(*e428*) showed the same phenotype as *zfp-1* and *lin-35* mutants (Figure 3B). In *dpy-21* loss of function mutants the X chromosome is transcriptionally upregulated [68]. However, a microarray study in *zfp-1*(*ok554*) and *lin-35*(*n745*) mutants did not detect an accumulation of X linked genes among the genes upregulated in these two mutant backgrounds. Furthermore, none of the core DCC components were significantly up- or downregulated in *zfp-1*(*ok445*) or *lin-35*(*n745*) mutants [69]. This clearly indicates that ZFP-1 and LIN-35 are not playing a direct role in dosage compensation per se. Nevertheless, it is certainly remarkable that in combination with *oxIs12*, mutations in *zfp-1, lin-35* and *dpy-21* behave similarly.

Taken together, our data show that the use of large multi-copy transgenes in *C. elegans* can be problematic, at least in combination with mutations in certain genes. Genetic screens are nowadays usually performed in sensitized backgrounds, which clearly increase the risk of finding candidates whose genetic link to the process under study is difficult to establish. In our case we were screening for axon guidance genes. However, our in-depth analysis of *zfp-1* and *lin-35* proved that they are clearly not “axon guidance” genes. Rather, loss of *zfp-1* or *lin-35* likely disrupts the normal gene expression pattern, indirectly leading to axon guidance defects. Which genes mediate this effect remains to be determined. The phenocopy of the *zfp-1* phenotype by the DCC mutant *dpy-21* suggests that at least one critical target gene is on the X chromosome.

A number of interesting questions remain open regarding the genes involved in the HSPG network and their function in dorsal axon guidance. Which signaling pathway is influenced by HSPGs? Is it Netrin signaling, the major dorsal guidance system or a parallel pathway such as Slit/Robo? What are the distinct functions and interaction partners of the two core proteins SDN-1 and LON-2? What is the role played by PTP-3 during dorsal axon guidance? What is the function of UNC-53 in cytoskeleton remodeling during dorsal axon guidance? Further biochemical experiments might provide answers to these questions.

## Supporting Information

Figure S1 **Estimation of the size of *oxIs12*.** Coverage analysis of the *lin-15AB* locus, which was used as co-injection marker to create the extrachromosomal array used for generation of *oxIs12*. Sequencing reads originating from the *lin-15AB* fragments in *oxIs12* will align at the endogenous *lin-15AB* locus and therefore lead to an increase of coverage in that area. While the average genome coverage was 70.35x, it increased to 11030x across the *lin-15AB* locus (indicated by red line). The part of the *lin-15AB* locus contained in the transgene can be determined precisely since coverage at the beginning and the end jumps from around 50x to over 4000x, and increases further from base to base (see enlargements of beginning and end).
(TIF)

Figure S2 **Effect of HSPG core protein and HS modifying enzyme mutants on D-type motor axon guidance.** Strains are ordered according to the severity of the defects. The first class contains all the single mutants as well as all the double mutants that show no/weak defects. The groups “weak enhancers” and “strong enhancers” are mostly the same strains as shown in Figure 1, additionally the strains containing *unc-52*(*e444*) are shown. Note that while *unc-52*(*e444*) weakly enhances *sdn-1*(*zh20*) it does not enhance the *lon-2*(*e678*) *sdn-1*(*zh20*) double mutant and is therefore considered not to play a role in D-type motor axon guidance. Grey bars represent the number of commissural axons growing away from the ventral nerve cord (VNC); white bars indicate the number of commissural axons reaching the DNC. Dashed lines indicate limits according to Figure 1A. Numbers are from 50 L1 animals +/- SEM. For statistical evaluation of relevant strains see Figure 1A.
(TIF)

Figure S3 **Outline of the screen.** Young adult P0 animals carrying either *hse-5*(*tm472*) or *sdn-1*(*zh20*) as a background mutation and the *oxIs12* transgene to label D-type motor neurons were mutagenized with EMS. Three P0 animals were put on one 9cm plate and transferred to fresh plates twice a day. F1 animals were allowed to lay eggs for about 12h before being removed from the plate. F2 animals were screened under a dissection fluorescence microscope for candidates phenocopying the strong D-type axon guidance defects of *hse-5*(*tm472*)*; sdn-1*(*zh20*) double mutants. Candidates were singled out and their progeny rescreened a couple of days later to confirm the phenotype.
(TIF)

Table S1 **Summary of all genes treated with RNAi.**
(DOC)
