# Correction: A Network of HSPG Core Proteins and HS Modifying Enzymes Regulates Netrin-Dependent Guidance of D-Type Motor Neurons in *Caenorhabditis elegans*

**DOI:** 10.1371/annotation/439d7826-5008-437d-9b41-5afb31c704a1

**Published:** 2014-01-17

**Authors:** Stephan Gysi, Christa Rhiner, Stephane Flibotte, Donald G. Moerman, Michael O. Hengartner

In Table 1, regarding the allele op468, the correct breakpoints are as follows: 

5': AAATCAATATTTCAGCAATCG

3': AAGAGTTCATTTAGCTGTAT

