# Supplementary figures and images for: A Network of HSPG Core Proteins and HS Modifying Enzymes Regulates Netrin-Dependent Guidance of D-Type Motor Neurons in Caenorhabditis elegans
Source: PLoS One. 2013 Sep 16;8(9):e74908. doi: 10.1371/journal.pone.0074908 (PMC3774775; doi:10.1371/journal.pone.0074908)

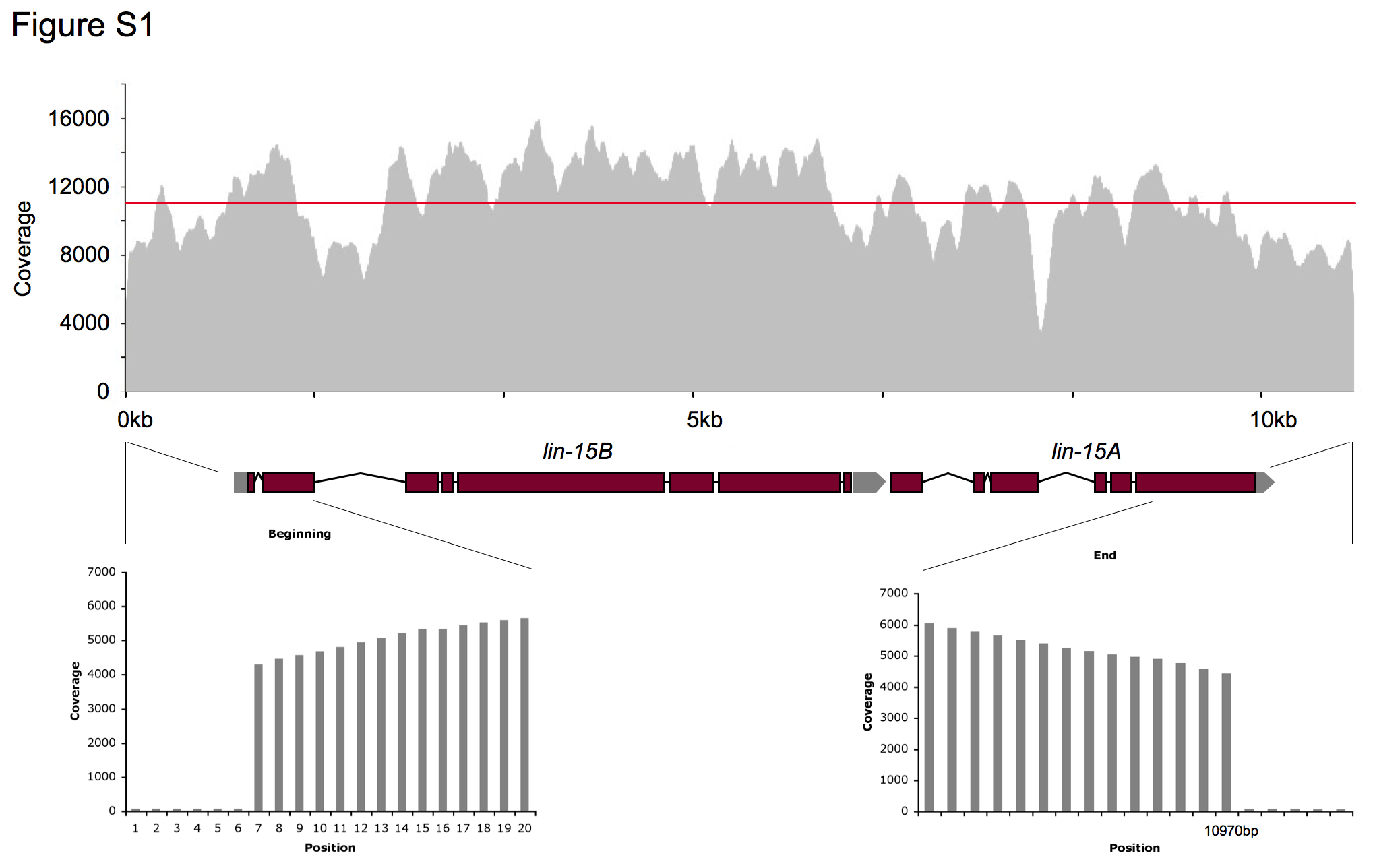

Supplement: Figure S1 — Estimation of the size of oxIs12. Coverage analysis of the lin-15AB locus, which was used as co-injection marker to create the extrachromosomal array used for generation of oxIs12. Sequencing reads originating from the lin-15AB fragments in oxIs12 will align at the endogenous lin-15AB locus and therefore lead to an increase of coverage in that area. While the average genome coverage was 70.35x, it increased to 11030x across the lin-15AB locus (indicated by red line). The part of the lin-15AB locus contained in the transgene can be determined precisely since coverage at the beginning and the end jumps from around 50x to over 4000x, and increases further from base to base (see enlargements of beginning and end). (TIF) [file pone.0074908.s001.tif]

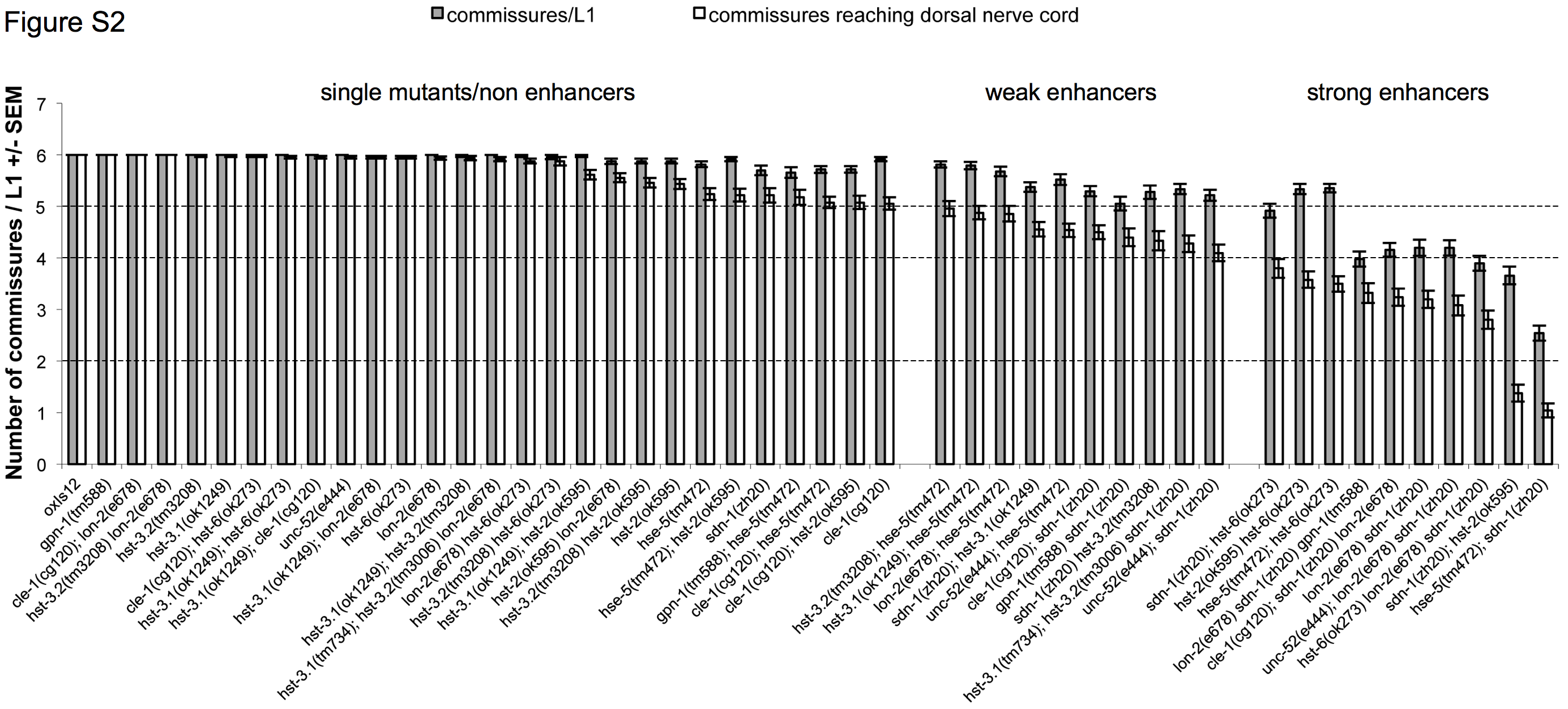

Supplement: Figure S2 — Effect of HSPG core protein and HS modifying enzyme mutants on D-type motor axon guidance. Strains are ordered according to the severity of the defects. The first class contains all the single mutants as well as all the double mutants that show no/weak defects. The groups “weak enhancers” and “strong enhancers” are mostly the same strains as shown in Figure 1, additionally the strains containing unc-52(e444) are shown. Note that while unc-52(e444) weakly enhances sdn-1(zh20) it does not enhance the lon-2(e678) sdn-1(zh20) double mutant and is therefore considered not to play a role in D-type motor axon guidance. Grey bars represent the number of commissural axons growing away from the ventral nerve cord (VNC); white bars indicate the number of commissural axons reaching the DNC. Dashed lines indicate limits according to Figure 1A. Numbers are from 50 L1 animals +/- SEM. For statistical evaluation of relevant strains see Figure 1A. (TIF) [file pone.0074908.s002.tif]

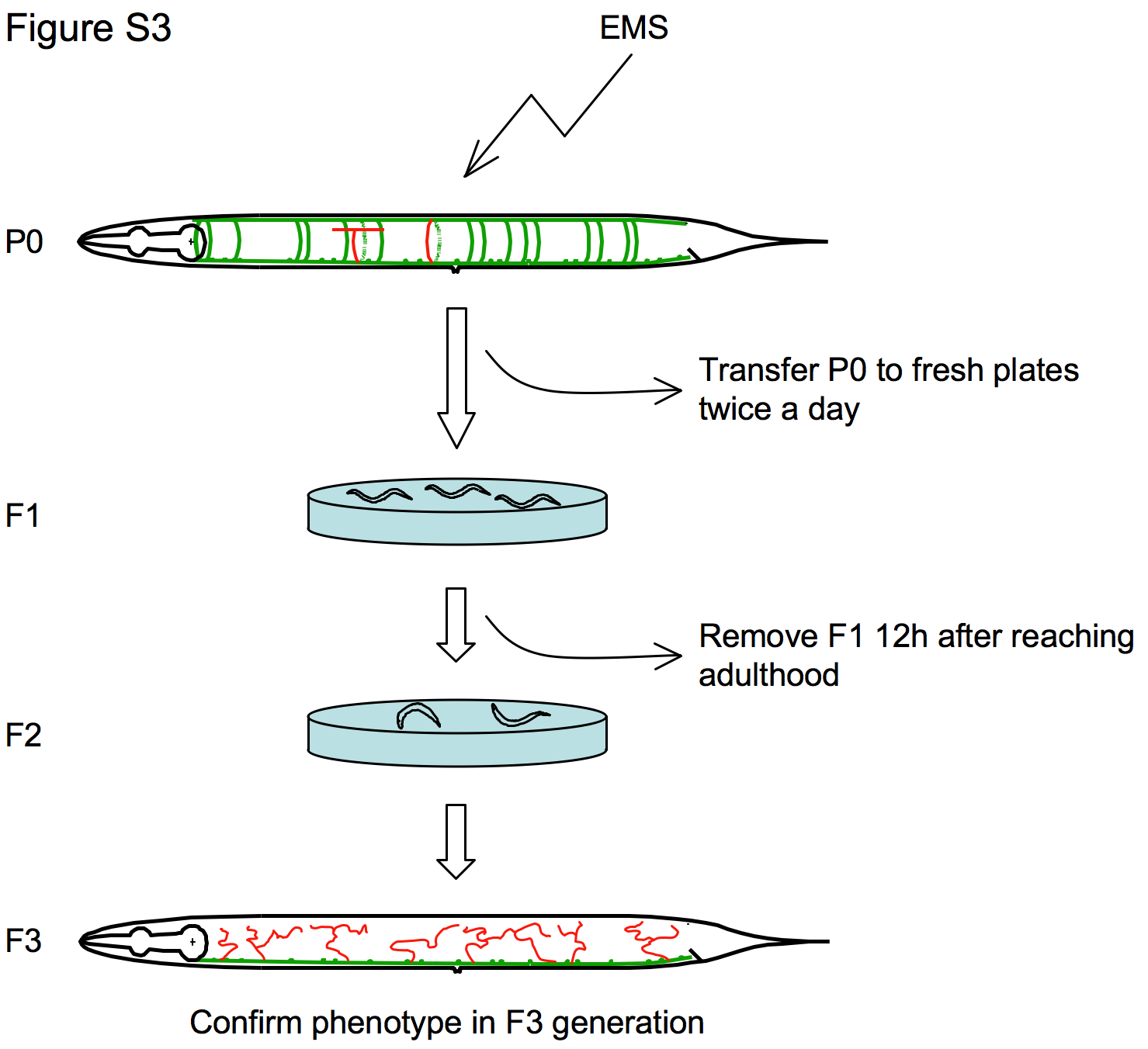

Supplement: Figure S3 — Outline of the screen. Young adult P0 animals carrying either hse-5(tm472) or sdn-1(zh20) as a background mutation and the oxIs12 transgene to label D-type motor neurons were mutagenized with EMS. Three P0 animals were put on one 9cm plate and transferred to fresh plates twice a day. F1 animals were allowed to lay eggs for about 12h before being removed from the plate. F2 animals were screened under a dissection fluorescence microscope for candidates phenocopying the strong D-type axon guidance defects of hse-5(tm472); sdn-1(zh20) double mutants. Candidates were singled out and their progeny rescreened a couple of days later to confirm the phenotype. (TIF) [file pone.0074908.s003.tif]
