# Supplementary material for: A Network of HSPG Core Proteins and HS Modifying Enzymes Regulates Netrin-Dependent Guidance of D-Type Motor Neurons in Caenorhabditis elegans
Source: PLoS One. 2013 Sep 16;8(9):e74908. doi: 10.1371/journal.pone.0074908 (PMC3774775; doi:10.1371/journal.pone.0074908)
Supplement: Table S1 — Summary of all genes treated with RNAi. (DOC) [file pone.0074908.s004.doc]

**Table S1: Summary of all genes treated with RNAi.**

| Gene name | Ahringer clone | Axon guidance defects | Comments |
| --- | --- | --- | --- |
|  |  |  |  |
| Argonaut genes^a^ |  |  |  |
| *F56A6.1/sago-2* | 1 D6 | - | Clone contains *C18E3.7*, will knock down both |
| *Y110A7A.18/ppw-2* | - | - | This study |
| *F55A12.1* | - | - | This study |
| *R06C7.1* | 11 C11 | - |  |
| *D2030.6/prg-1* | - | - | This study |
| *C04F12.1* | - | - | This study |
| *T23D8.7* | 17 B1 | - |  |
| *T07D3.7/alg-2* | 33 C3 | - |  |
| *Y49F6A.1* | - | - | This study |
| *ZK1248.7* | 45 G3 | - |  |
| *F58G1.1* | - | - | This study |
| *C14B1.7* | 69 E2 | - |  |
| *C16C10.3* | 70 C6 | - |  |
| *ZK757.3/tag-76* | 83 C8 | - |  |
| *M03D4.6* | 100 E7 | - |  |
| *C01G5.2/prg-2* | 100 H6 | - |  |
| *F20D12.1/csr-1* | 104 C1 | - |  |
| *T22B3.2* | - | - | This study |
| *T22H9.3* | 125 D12 | - |  |
| *R09A1.1/ergo-1* | 127 C6 | - |  |
| *K12B6.1/sago-1* | 139 F7 | - |  |
| *K08H10.7/rde-1* | 148 H11 | - |  |
| *ZK218.8* | 166 H10 | - |  |
| *R04A9.2/nrde-3* | 176 A9 | - |  |
| *F48F7.1/alg-1* | 198 B8 | - |  |
| *C18E3.7/ppw-1* | - | - | *C18E3.7/ppw-1* is more or less identical to *sago-2* |
| *C06A1.4* | - | - | *C06A1.4* is a pseudogene very similar to *F58G1.1* |
|  |  |  |  |
| down regulated in *zfp-1(ok554)* and *lin-35(n745)*^b^ |  |  |  |
| *K11G9.4/egl-46* | 140 F1 | - |  |
| *K12F2.2/vab-8* | 153 H3 | - |  |
| *F02G3.1/ncam-1* | 176 E9 | - |  |
| *F22D3.6* | 48 C12 | - |  |
| *C04F5.3/unc-46* | 137 C4 | - |  |
| *VC5.2* | 141 E3 | - |  |
| *Y37D8A.23/unc-25* | 87 H12 | - |  |
|  |  |  |  |
| SynMuv A^c^ |  |  |  |
| *B0454.1/lin-8* | 38 F9 | - |  |
| *ZK678.1/lin-15a* | - | - |  |
| *lin-38* | - | - |  |
| *ZK673.3/lin-56* | - | - |  |
| *K12C11.2/smo-1* | 1 H7 | ++ (very sick) |  |
| *W02A11.4/uba-2* | 23 A8 | ++ |  |
|  |  |  |  |
| SynMuv B^c^ |  |  |  |
| *T23G7.1/dpl-1* | 53 D7 | - |  |
| *Y102A5C.18/efl-1* | 166 D11 | - |  |
| *W07B3.2/gei-4* | 66 E7 | - |  |
| *C53A5.3/hda-1* | 159 C6 | - |  |
| *K01G5.2/hpl-2* | 85 A1 | - |  |
| *F26F12.7/let-418* | 138 H8 | - |  |
| *ZK637.7/lin-9* | 81 B4 | - |  |
| *C03B8.4/lin-13* | - | - |  |
| *ZK662.4/lin-15b* | - | - |  |
| *C32F10.2/lin-35* | 7 G8 | - |  |
| *F44B9.6/lin-36* | 79 A5 | - |  |
| *ZK418.4/lin-37* | 77 A4 | - |  |
| *ZK632.13/lin-52* | 83 B12 | - |  |
| *K07A1.12/lin-53* | 16 B7 | - |  |
| *JC8.6/lin-54* | 116 C5 | + |  |
| *R06C7.7/lin-61* | 11 D5 | - |  |
| *Y71G12B.9/lin-65* | 29 G10 | - |  |
| *M04B2.1/mep-1* | 112 F5 | - |  |
| *R05D3.11/met-2* | 79 H12 | - |  |
| *F26G5.9/tam-1* | 137 A4 | - |  |
| *F53B3.1/tra-4* | 180 A9 | + |  |
| *F29B9.6/ubc-9* | 97 F3 | + |  |
| *E01A2.4* | 4 B10/11 | - |  |
| *W01G7.3/rpb-11* | 63 A12 | - |  |
|  |  |  |  |
| SynMuv C^c^ |  |  |  |
| *Y111B2A.11/epc-1* | 87 E7 | + |  |
| *VC5.4/mys-1* | 141 E5 | - |  |
| *Y111B2A.22/ssl-1* | - | - |  |
| *C47D12.1/trr-1* | 58 G10 | - |  |
|  |  |  |  |
| RTK-Ras^c^ |  |  |  |
| *C01C7.1/ark-1* | - | - |  |
| *T24C12.2/gap-1* | - | - |  |
| *M02A10.3/sli-1* | 176 E12 | - |  |
|  |  |  |  |
| SynMuv Sup., chr. remod.^d^ |  |  |  |
| *C34B7.4/mys-4* | 13 D11 | - |  |
| *M04B2.3/gfl-1* | 112 F7 | - |  |
| *Y37D8A.9/mrg-1* | 87 G12 | - |  |
| *Y105E8A.17/ekl-4* | - | - |  |
| *ZK1127.3* | 48 E4 | - |  |
| *C08B11.6* | 50 G8 | - |  |
| *C17E4.6* | 15 F8 | - |  |
| *CD4.7* | 138 D5 | - |  |
| *C14B1.4/tag-125* | 69 D11 | - |  |
| *ZK863.6/dpy-30* | 153 G9 | - |  |
| *C46A5.9/hcf-1* | 103 G4 | - |  |
| *Y2H9A.1/mes-4* | 156 E7 | - |  |
| *F37A4.8/isw-1* | 76 C8 | - |  |
| *C01G8.9/lss-4* | 6 E9/10 | - |  |
| *C34D4.14* | 102 C2 | - |  |
| *F30A10.10* | 15 H5 | - |  |
| *B0205.3/rpn-10* | 18 G4 | - |  |
| *ZK20.5/rpn-12* | 58 G8 | - |  |
| *R08C7.3/htz-1* | 97 B11 | - |  |
| *T02C12.3* | 70 A11 | - |  |
| *ZK856.9/tag-143* | 149 D11 | - |  |
| *F52B11.1* | 117 G1 | - |  |
| *Y53G8AR.2* | 90 H6 | - |  |
| *B0035.4/pfd-4* | 112 B8 | - |  |
| *F54F2.2/zfp-1* | 80 H9 | ++++ |  |
| *F02E9.4/pqn-28* | 13 F2 | - |  |
| *C06E7.1* | 100 A11 | - |  |
| *C34E10.8* | 73 A10 | - |  |
| *F54D11.2* | 136 A12 | - |  |
| *K08F4.2* | 109 D9 | - |  |
| *M03C11.3* | 84 D2 | - |  |
| *T07E3.3* | 76 F1 | - |  |
|  |  |  |  |
| lysine demethylases^e^ |  |  |  |
| *ZK593.4/rbr-2* | - | - | This study |
| *Y40B1B.6/spr-5* | 24 D1 | - |  |
| *C29F7.6* | 197 C11 | - |  |
| *F23D12.5* | 199 A3 | - |  |
| *D2021.1/utx-1* | 189 E5 | + |  |
| *F18E9.5/tag-279* | 189 F1 | - |  |
| *F29B9.2* | 97 E12 | - |  |
| *Y48B6A.11/jmjd-2* | - | - | This study |
|  |  |  |  |
| LSD1 homolog^f^ |  |  |  |
| *Y40B1B.6/spr-5* |  | - |  |
| *T08D10.2/lsd-1* |  | - |  |
|  |  |  |  |
| H3K4 methyl transferase^g^ |  |  |  |
| *C26E6.9/set-2* | 72 D5 | - |  |
| *T12D8.1/set-16* | 89 B6 | - |  |
| *Y17G7B.2/ash-2* | - | nd |  |
| *F52B11.1* | 117 G1 | - |  |
| *F21H12.1/rbbp-5* | 46 C8 | - |  |
| *C33H5.6/swd-2.1* | 103 G9 | - |  |
| *C33H5.7/swd-2.2* | 103 G10 | + |  |
| *C14B1.4/wdr-5.1* | 69 D11 | - |  |
| *ZK863.6/dpy-30* | 153 G9 | - |  |
|  |  |  |  |
| hDOT1 homolog |  |  |  |
| *Y39G10AR.18* | 27 C9 | - |  |
| *ZC53.6* | 178 F12 | - |  |
| *D1053.2* | 194 E5 | - |  |
| *F54F7.7* | 194 G2 | - |  |
| *F55G7.2* | - | - | This study |
| *W06D11.4* | 195 E3 | - |  |
|  |  |  |  |
| Rpd3 homolog^i^ |  |  |  |
| *C53A5.3/hda-1* | 159 C6 | - |  |
| *R06C1.1/hda-3* | - | nd |  |
| *C08B11.2/hda-2* | 50 G4 | - |  |
| *F41H10.6/hdac-6* | 99 C12 | - |  |
| *C10E2.3/hda-4* | 202 A11 | - |  |
| *Y51H1A.5/hda-6* | 62 G3 | - |  |
| *C35A5.9/hdac-11* | - | nd |  |
| *F43G6.11* | 59 B2 | - |  |
|  |  |  |  |
| Htz1 homolog^i^ |  |  |  |
| *R08C7.3/htz-1* | 97 B11 | - |  |
| *C50F4.13/his-35* | - | nd |  |
| *T23D8.6/his-68* | 17 A12 | - |  |
| *ZK1251.1/htas-1* | 108 A12 | - |  |
|  |  |  |  |
| Sas2 homolog^i^ |  |  |  |
| *K03D10.3/mys-2* | - | nd |  |
| *VC5.4/mys-1* | 141 E5 | + |  |
| *C34B7.4/mys-4* | 13 D11 | - |  |
| *R07B5.9/lsy-12* | 148 F2 | - |  |

Gene name: given are sequence name/main name according to www.wormbase.org. Function: indicates why gene has been chosen for knock down; superscript is giving the reference or a more precise description. Ahringer clone: clone nr of the clone corresponding to the gene. Clones were confirmed to contain the right sequence by sequencing; - means that the gene was either not contained in the library or the clone contained a wrong sequence. Axon guidance defect: D-type axon guidance defects in: + = 25%; ++ = 50%; +++ = 75%; ++++ = 100% of the animals; nd = not determined. *eri-1(mg366); sdn-1(zh20) oxIs12* animals were submitted to feeding RNAi as described in Materials and Methods. ^a^: [S1]; ^b^: [68]; ^c^: [63]; ^d^: [36]; ^e^: lysin demethylases, wormbase search; ^f^: LSD1 protein blast; ^g^: [S2]; ^h^: hDOT1 blast, gene; ^i^: [S3].

REFERENCES

[S1] Yigit E, Batista PJ, Bei Y, Pang KM, Chen CC, *et al.* (2006) Analysis of the *C. elegans* Argonaute family reveals that distinct Argonautes act sequentially during RNAi. Cell 127: 747-57.

[S2] Simonet T, Dulermo R, Schott S, Palladino F (2007) Antagonistic functions of SET-2/SET1 and HPL/HP1 proteins in *C. elegans* development. Dev Biol 312: 367-83.

[S3] Verzijlbergen KF, Faber AW, Stulemeijer IJ, van Leeuwen F (2009) Multiple histone modifications in euchromatin promote heterochromatin formation by redundant mechanisms in *Saccharomyces cerevisiae*. BMC Mol Biol 10:76.

single mutants/nonenhancers
